# Prevention and the treatment of oral mucositis: the efficacy of sodium bicarbonate vs other agents: a systematic review

**DOI:** 10.1186/s12903-022-02586-4

**Published:** 2023-01-03

**Authors:** O. Di Fede, F. Canepa, L. Maniscalco, P. Tozzo, D. Matranga, G. Giuliana

**Affiliations:** 1 Department of Surgical, Oncological and Oral Sciences (Di.Chir.On.S.), University of Palermo, Via L. Giuffrè 5, 90127 Palermo, Italy; 2 Department of Health Promotion, Mother and Child Care, Internal Medicine and Medical Specialties, University of Palermo, Palermo, Italy; 3 U.O.C. of Stomatology, Ospedali Riuniti “Villa Sofia-Cervello” of Palermo, Palermo, Italy

**Keywords:** Oral mucositis, Baking soda, Sodium bicarbonate

## Abstract

**Introduction:**

Oral mucositis (OM) is a major side effect of cancer therapy, which is associated with significant symptoms, treatment delays and increased costs for the health system. It is an important component of the quality of life of cancer patients and, until now, there has been no gold standard regarding prevention or treatment of this pathology. Notwithstanding the paucity of treatment guidelines (due to limited evidence from high-quality, rigorous studies), sodium bicarbonate (SB) rinses are one of the most used agents for OM management.

**Objectives:**

A systematic review (2000–2022) was performed in order to compare and examine different agents versus sodium bicarbonate (SB) in preventing or treating OM.

**Sources:**

Eleven randomized controlled trials (RCT) were evaluated: four were conducted for the prevention and seven for the management of OM. The risk of bias of RCTs was assessed using the revised Cochrane risk of bias tool for randomized trials.

**Study selection:**

According to the RoB2 evaluation for randomized trials, four RCTs were judged to be at a high risk of bias, two were rated as ‘problematic’, while five were deemed to be a low risk of bias.

**Conclusions:**

The results revealed that there was no evidence for supporting SB in OM treatment regarding management and prevention.

**Clinical significance:**

Results showed in this review takes on a strategic importance in the use of SB for OM management or prevention; indiscriminate use of SB could be counterproductive because it causes a sudden pH increase and it delays proper OM pharmacological treatment.

## Introduction

Oral mucositis (OM) is a significant side effect of cytotoxic anti-cancer chemotherapy, and head and neck radiotherapy (RT) [1]. It occurs in approximately 30–40% of cancer patients, who are treated with chemotherapy (e.g. antimetabolites 5-fluorouracil, methotrexate, and cytarabine). This percentage rises to 60–85% for patients undergoing a hematopoietic stem cell transplantation, and to almost 90% for head and neck cancer patients, who are treated with radio- and chemotherapy [2].

Of the 1.8 million cancer patients in the USA, almost half will suffer a degree of mucositis. For many patients, OM caused by chemotherapy (CT) will be of such severity so as to cause major diet modifications and weight loss: opioid analgesics and supplemental nutrition will be required in order to avoid disrupting optimal cancer therapy. Furthermore, chemotherapy mucositis can induce a superinfection, with the additional threat of bacteraemia and sepsis. Patients with OM are more likely to have negative treatment outcomes, a poorer quality of life, and incur higher financial costs than patients who do not develop this condition.

CT-associated OM (CT-OM) originates in the submucosa layer and it becomes to be clinical visible nearly 4 days after first CT infusion; typical primary manifestations are redness, atrophy, and sensitivity. The degeneration continues, and ulceration occurs a few days later, persisting for 1–2 weeks, after which it typically heals spontaneously. This phase, the ulcerative one, is the most painful and it is correlated with poor health resolution. The sequelae of CT-OM, which include pain, odyno/dysphagia, dysgeusia, decreased oral intake and systemic infection, frequently require treatment delays, interruptions, and suspension, all of which not only negatively impact the quality of life but also tumor control and survivorship.

Recently, CT-OM onset has been identified as a five phase model but clinically, OM presents itself at the fourth phase of the inflammation process, that is, the ulceration phase, evidently compromising mucosae integrity with patients complaining of pain [2].

OM is often reported only when a high-grade mucositis develops, necessitating clinical treatment. This is determinant for OM epidemiological data, which are still considered to be underestimated and contradictory in the absence of a gold standard scale, with which to score severity. Typically, patients are not screened by trained oral specialists. To date, different scales for grading mucositis have been identified with varying parameters. Oral assessment is essential for the comparing management strategies for OM. Table 1 provides a list of some the most used scoring list in adult patients. Of the pediatric population, severe OM can appear from the first week of chemotherapeutic treatment with its incidence increasing over time. The fifth week of treatment has the highest incidence of severe OM, which is detected in typical oral sites. Children and adolescents are more prone to develop severe OM, with younger children having a greater probability of an occurrence of CT-induced severe OM (90%) among children under 12 years old) [3]. Pediatric patients with hematologic tumors are more likely to develop severe OM than those with solid tumors [4].

**Table 1:** OM scoring list in adult patients

Scale	Description
World Health Organization (WHO) [5]	Combined objective (ulcers and erythema) and function. Scale 0 to 4
Oral Mucositis Assessment Scales (OMAS) [6]	Erythema and ulceration at 9 sites
Oral Assessment Guide (OAG) [7]	Objective (erythema), symptoms (pain, salivary change) and function
Eastern Cooperative Oncology Group (ECOG) [8]	Mucositis severity is differently classified based on the anatomic site of development
Common Terminology Criteria for Adverse Event (CTCAE) [9]	Mucositis severity measure scale based on anatomic site of development and on the kind of treatment, either chemo or radiotherapy
MacDibbs mouth assessment [10]	14-item instrument grouped into four sections: the patient information section includes seven items that measure the patient’s oral symptoms including problems with pain, dryness, eating, talking, swallowing, tasting and saliva production

There is a paucity of data in the literature focusing on child OM assessment: the Children’s International Mucositis Evaluation Scale (ChIMES) [5], OMAS and OAG are possible contenders albeit with poor results [6]. Therefore, a focus on the approach to child OM assessment is required, including a: feasible light source, mechanisms to ensure optimal visualization of the oral cavity, and training in dealing with the challenging behaviour of children. These factors constitute a challenge in the management of OM in children.

In order to prevent and manage OM, many protocols have been proposed in the literature even if their efficacy is still a matter of debate. Numerous natural and pharmacological agents have been tested with poor results. However, there is limited evidence from reliable studies with numerous heterogeneous protocols characterizing the various studies, making it difficult to draw conclusions. This heterogeneity can be attributed to the agents used in the protocols, in addition to differences in terms of timing, frequency, intensity, equipment, and storage conditions. All of these are contributing factors in reproducing a protocol for comparison [7].

Whilst several treatment options are available, there is no clear consensus regarding prevention, especially for young people. Current recommendations for the management of OM are very limited, and the standard of care for this complication has hitherto been palliative [8]; the aim of OM management is to control symptoms via topical or systemic analgesics and the topical application of barrier agents, thereby covering injured mucosae as a salve or ointment..

Evaluating clinical practice guidelines, Sung et al. reported their findings regarding cryotherapy, lower level light therapy (LLLT) and keratinocyte growth factor (KGF) as an OM prevention tool, however, with unconvincing recommendations for use [9].

In a recent review Fent et al. demonstrated that oral administration of probiotics could decrease OM incidences and chemotherapy-induced diarrhea [10].

In their systematic review Daugelaité et al. demonstrated the following as the most effective treatment or/and prevention methods for treating OM: laser therapy, cryotherapy, professional oral hygiene, antimicrobial agents, Royal jelly, *Lactobacillus brevis* lozenges, zinc supplementation and benzydamine. However, it should be stressed that controversial results have been obtained in various studies, which have examined the same treatment or prevention method [11].

According to the literature, the latest version of the MASCC/ISOO Clinical Practice Guidelines for the Management of Mucositis Secondary to Cancer Therapy suggests that the implementation of multi-agent combination oral care protocols (basic oral care) is beneficial for the prevention of OM during chemotherapy, head and neck radiation therapy, and hematopoietic stem cell transplantation [12]. Specifically, Sodium Bicarbonate (SB), a component of basic oral care, is one the most used agents regarding the prevention and treatment of OM. It is also used as a cleansing agent due to its ability to dissolve mucus and loosen debris [13]. The benefits of SB use is due to its alkalizing effect (thereby raising oral pH), which prevents the growth of aciduric bacteria, making saliva more fluid and preventing the accumulation of detritus [14,15]. Furthermore, its use is strongly encouraged when considering other factors such as low cost, no side effects, patient-friendly application, and its long shelf life [25]. The combination regimen of CHX, SB and nystatin has been documented to be one of the many standard protocols developed for patients undergoing chemotherapy, as reported in the clinical study conducted by Mubaraki et al. [16].

The MASCC/ISOO Clinical Practice Guidelines do not refer to the use of saline or SB rinses in the prevention or treatment of OM-CT in patients undergoing cancer therapy due to the limited data regarding saline and SB. However, despite this limited data, the panel recognizes that these are inert, bland rinses which increase oral clearance, which may help to maintain oral hygiene and enhance patient comfort. Basic oral care remains an important best practice for patients undergoing cancer treatment [12]. The aim of this study is, therefore, to conduct a systematic review examining and comparing different agents used to prevent or treat OM versus SB.

## Materials & methods

This present study followed the *Preferred Reporting Items for Systematic Reviews and Meta-analyses* (PRISMA) and the *Meta-analysis of Observational Studies in Epidemiology* (MOOSE) guidelines. The PICO search strategy considers the comparison of different OM prevention/management protocols; selected outcomes are *incidence* and *the severity of OM*.

### Evidence gathering

Ideally, the review process should be well developed and planned in order to reduce biases and eliminate irrelevant and poor quality studies. The steps for implementing a systematic review include: (i) carefully formulating the clinical question to be answered (PICO); (ii) developing a protocol (inclusion and exclusion criteria); (iii) performing a detailed and exhaustive literature search; and (iv) screening the study abstracts, which have been identified in the search and subsequently the selected complete texts (PRISMA).

### Eligibility criteria

Only randomized clinical trials with human patients have been included.

### Information sources and search strategy

A systematic, electronic search through different, biomedical databases (e.g. PubMed, Ovide/MEDLINE, Web of Knowledge, Embase and the Cochrane Library) was performed by two authors (F.C. and O.D.F.) in the period from January 2000 to May 2022. This search was restricted to abstracts in English. The MeSH searched terms were “Stomatitis” and “Sodium Bicarbonate”, and open research regarding “baking soda mucositis” was also performed. Other data sources (from international meetings and indexed dentistry journals such as the *Journal of Dentistry, Journal of Oral Maxillofacial Surgery, Journal of Dental Research*) were also scanned as sources of grey literature*.*

### Study selection

Screening and eligibility were assessed independently by two reviewers (F.C. and O.D.F.), who were in agreement regarding the results. The titles and abstracts were initially screened for relevance and possible eligible results, and the full texts were thereafter retrieved. Finally, the reviewers combined the results to create a corpus of selected papers in order to assess for final eligibility. Tables 2 and 3 summarize the eligible studies, which have been divided into *prevention* and *therapy* (according the purpose of the study) and their principal features.

**Table 2:** Eligible studies for OM prevention

First author (year)	Population	Intervention	Experimental arm 1	Experimental arm 2	Control arm	Outcomes	Study design	OM assessment grade	OM assessment performer	Results
Piredda (2017) [17]	60 patients under chemotherapy (30 in the experimental arm and 30 in the control arm)	prevention	dry extract of propolis with 8–12% of galangin plus mouth rinsing with SB	–	SB mouthwash	Clinical efficacy, safety, tolerability and compliance of propolis, incidence and severity of OM	RCT	Modified National Cancer Institute Scale version 4.0	Unknown plus telephone assessment	Propolis plus SB was more effective than SB alone in preventing OM graded higher than G1.
Chitapanarux (2018) [18]	60 patients under chemo + radiotherapy (30 in the experimental arm and 30 in the control arm)	prevention	0.15% benzydamine hydrochloride mouthwash	–	SB mouthwash	Effectiveness of the mouthwash, pain score	RCT	The Oral Mucositis Assessment Scale (OMAS)	Oncologists	The median of OMAS scores at every weekly assessment was lower in the benzydamine HCl group compared to that of the control group.
De Sanctis (2019) [19]	68 patients under chemo + radiotherapy (32 in the experimental arm and 36 in the control arm)	prevention	*Lactobacillus brevis* CD2 lozenges	–	SB mouthwash	Incidence of OM, pain, dysphagia, body weight loss and quality of life	RCT	NCI Common Toxicity Criteria scoring system version 4.0	Trained physicians	No differences
Alkhouli (2021) [20]	22 patients under chemotherapy (11 in the experimental arm and 11 in the control arm)	prevention	70% aloe-vera solution	–	SB 5% mouthwash	Efficacy of *Aloe Vera*, severity of mucositis	RCT	Who grading scale	unknown	Topical use of aloe-vera was efficient in the prevention of OM compared to SB 5%.

**Table 3:** Eligible studies for OM therapy

First author (year)	Population	Intervention	Experimental arm 1	Experimental arm 2	Control arm	Outcomes	Study design	OM assessment grade	OM assessment performer	Results
Dodd (2000) [21]	142 patients under chemotherapy (51 in the experimental arm 1, 49 in the experimental arm 2, 42 in the control arm)	therapy	CHX gluconate mouthwash (0.12%)	“Magic” mouthwash: lidocaine solution (0.5%), 5 mL; diphenhydramine hydrochloride, 0.25 mL; and aluminum hydroxide suspension, 14.75 mL	Salt and SB mouthwash	Effectiveness of the mouthwash, severity of OM	RCT	Oral assessment guide	Intervention nurses plus telephone assessment	No difference with a systematic oral hygiene protocol.
Dodd (2003) [22]	30 patients under radiotherapy (14 in the experimental arm and 16 in the control arm)	therapy	Micronized sucralfate (Carafate) mouthwash	–	Salt and SB mouthwash	Severity of OM, severity of mucositis-related pain, and time required to heal RT-induced mucositis	RCT	MacDibbs score	Intervention nurses plus telephone assessment	No differences
Satheeshkumar (2010) [23]	24 patients under radiotherapy (12 in the experimental arm and 12 in the control arm)	therapy	Triclosan mouthwash	–	SB mouthwash	Severity of OM, food intake, weight loss, and pain	RCT	Who grading of mucositis	unknown	Control group took 45 days to resolve; experimental group took less than 28 days.
Choi (2012) [24]	48 patients under chemotherapy (24 in the experimental arm and 24 in the control arm	therapy	CHX mouthwash and SB	–	SB mouthwash	Effectiveness of oral care by SB solution and CHX mouthwash on the severity of OM, limitations of daily activities, oral bacterial colonization, and clinical signs associated with infection	RCT	WHO Oral Toxicity Scale (WHO, 1979)	unknown	The incidence rate of ulcerative OM in the SB group (25.0%) was significantly lower than that in the CHX group (62.5%).
Cabrera-Jaime (2017) [25]	45 patients under chemotherapy (15 in the experimental arm 1, 15 in the experimental arm 2, 15 in the control arm)	therapy	SB 5% aqueous solution plus *Plantago major* extract	SB 5% aqueous solution plus CHX 0.12%	SB 5% aqueous solution plus SB 5% aqueous solution	Mouthwash efficacy; pain intensity, oral intake capacity; and quality of life	RCT	WHO mucositis scale	Clinical nurse plus telephone assessment	No significant differences
Mubaraki (2020) [16]	45 patients under radiotherapy (15 in the experimental arm 1, 15 in the experimental arm 2, 15 in the control arm)	therapy	0.12% CHX gluconate + SB 3% aqueous solution + nystatin 5000 U/mL and supersaturated calcium phosphate rinse	0.12% CHX gluconate + SB 3% aqueous solution + nystatin 5000 U/mL and an extra-soft toothbrush	0.12% CHX gluconate + SB 3% aqueous solution + nystatin 5000 U/mL	Severity of OM	RCT	Who mucositis scale	Oncologic team	No differences
Mohammadi (2022) [26]	144 patients under chemotherapy (48 in each group)	therapy	Zinc chloride mouthwash 0.2%	SB mouthwash 5%	Sterile water mouthwash	Severity of OM, quality of life	RCT	WHO grading of mucositis	Clinical nurse in oncology clinic	The severity of OM in the SB and zinc chloride groups decreased from end of the first week until the third week. The zinc chloride group had better performance promoting the quality of life than the SB group.

### Data collection process

Data collection was independently performed by one author (F.C.), and the results were reviewed by a second author (O.D.F.) to verify their accuracy.

## Results

**Fig. 1:**
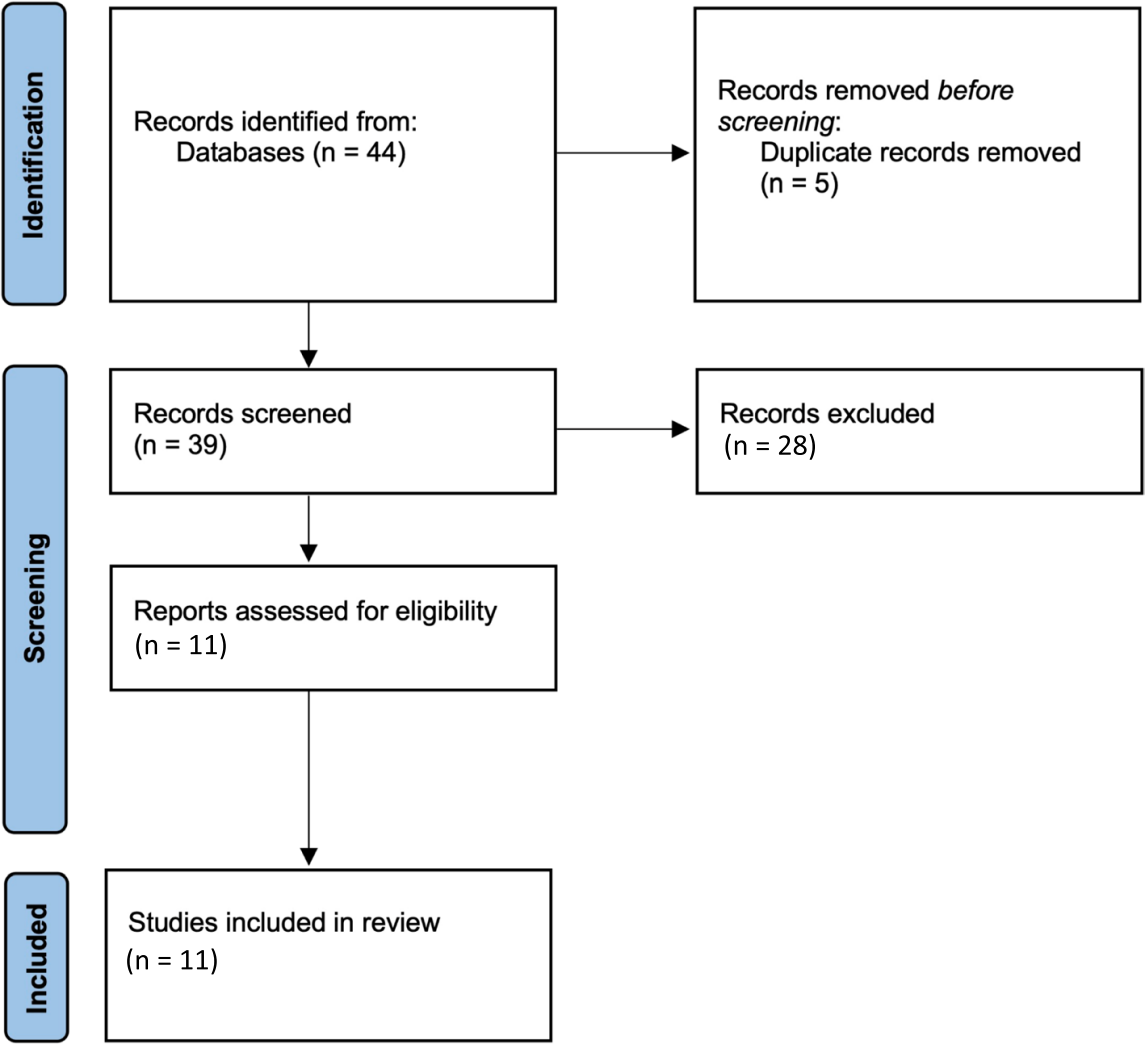
PRISMA flow diagram of the pooled-including studies

The PRISMA flow diagram in Fig. 1 describes the pooled-including studies. The initial search strategy identified 44 records, which were obtained by database searching. Two reviewers (F.C. and O.D.F.) independently screened the titles and abstracts to obtain a total of 39 articles (5 duplications were excluded). Of these 39 articles, 28 did not meet the inclusion criteria for this review, thus 11 articles were deemed to be *eligible*. Of the eleven randomized controlled trials (RCT), in which SB was compared to other agents, four trials had been performed for the prevention of OM and seven for its OM. The prevention agents were propolis, benzydamine hydrochloride, *lactobacillus brevis* CD2 and aloe-vera versus SB mouthwash.

Piredda et al. compared dry extract of propolis with 8–12% of galangin plus mouth rinsing with SB versus simple SB mouth rinsing in 60 patients undergoing chemotherapy. The results demonstrated that propolis plus SB was more effective than SB alone in preventing OM, which was graded to *exceeding G1* [17]. Chitapanarux et al. evaluated the effectiveness of a 0.15% benzydamine hydrochloride mouthwash versus a SB mouthwash in 60 patients undergoing chemo- and radiotherapy. The results indicated that the median OMAS scores at every weekly assessment were lower in the benzydamine HCl group, compared to that of the control group (SB) [18]. However, no differences were found in the study by De Sanctis et al. RCT, in which the use of *Lactobacillus brevis* CD2 lozenges was compared to SB mouthwash in 68 patients undergoing chemo- and radiotherapy [19]. A positive outcome for the topical application of aloe-vera was observed in the clinical study by Alkhouli et al. 22 patients undergoing chemotherapy were divided into two arms in order to compare the efficacy of 70% aloe-vera solution versus SB 5% mouthwash. The results demonstrated that aloe-vera was effective in the prevention of OM, when compared to SB 5% [20].

Approximately seven articles, evaluating the efficacy of several agents for OM treatment in the 2000 study by Dodd et al., compared CHX gluconate mouthwash 0.12% (group 1) versus a combined mouthwash of lidocaine solution 0.5%, diphenhydramine hydrochloride and aluminum hydroxide suspension (group 2) versus salt and soda mouthwash (group 3). This study involved 142 patients undergoing chemotherapy; the results revealed no differences when compared to a systematic oral hygiene protocol [21]. In 2003, Dodd e al. evaluated micronized sucralfate (Carafate) mouthwash versus salt and SB mouthwash in 30 patients undergoing radiotherapy with no differences in the results [22]. Satheeshkumar et al. evaluated the effect of triclosan mouthwash versus SB mouthwash in 24 patients undergoing radiotherapy. The final data confirmed that triclosan had a greater effect in treating OM (28 days out of the 45 days, required for the SB mouthwash) [23].

Choi et al. recruited 48 patients being treated with chemotherapy in order to compare a mixture of CHX and SB mouthwash versus SB mouthwash alone. The results revealed that the incidence rate of ulcerative OM in the SB group (25.0%) was significantly lower than in the CHX group (62.5%) [24]. However, no significant differences were observed in in the RCT study by Cabrera-Jaime et al., in which 45 patients undergoing chemotherapy were divided into three arms and tested for: SB 5% aqueous solution plus *Plantago major* extract, SB 5% aqueous solution plus CHX 0.12% or SB 5% aqueous solution plus SB 5% aqueous solution [25].

In 2020 Mubaraki et al. compared three different treatments in 45 patients being treated with radiotherapy. Group 1 used: 0.12% CHX gluconate + SB 3% aqueous solution + nystatin 5000 U/mL and supersaturated calcium phosphate rinse; group 2 used 0.12% CHX gluconate + SB 3% aqueous solution + nystatin 5000 U/mL and an extra-soft toothbrush; and group 3 used 0.12% CHX gluconate + SB 3% aqueous solution + nystatin 5000 U/mL. The results demonstrated no differences in the severity of OM patients [16]. Finally, Mohammadi et al. evaluated 144 a total of 3 groups of patients undergoing chemotherapy, including the use of: zinc chloride mouthwash 0.2%, SB mouthwash 5% and sterile water mouthwash. The severity of OM in the SB and zinc chloride groups decreased from the end of the first week until the third week but the zinc chloride group performed better, a fact promoting the quality of life, unlike the SB group [26].

### Risk of bias assessment

**Fig. 2:**
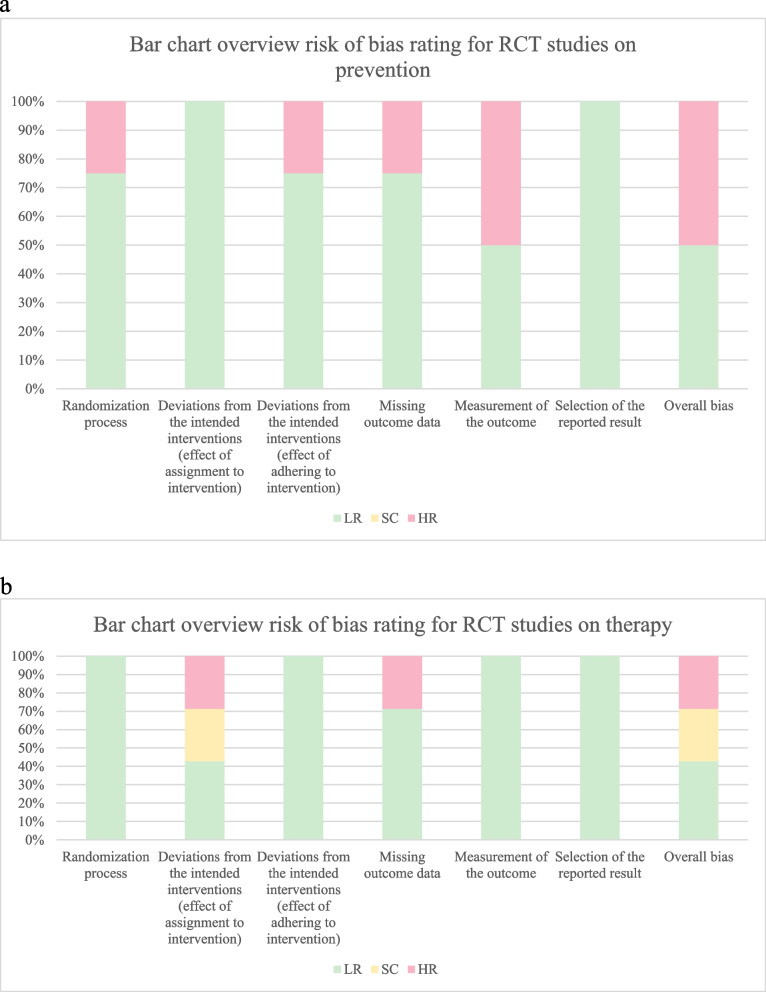
**a** Bar chart overview risk of bias evaluation for the selected RCT studies on prevention. **b** Bar chart overview risk of bias evaluation for the selected RCT studies on therapy

The potential bias of RCTs, using the revised Cochrane risk of bias tool for randomized trials [27,28], was assessed. All risk bias assessments were conducted by two reviewers (L.M., and D.M.). The RCT studies were judged for: bias arising from the randomization process, deviations from the intended interventions (defined as the effect of intervention assignment and the effect of adhering to intervention), an omission of outcome data, the measurement of the outcome, and the selection of the reported result. According to the RoB2 evaluation for randomized trials, four RCTs were judged to be at an elevated risk of bias (De Sanctis, Cabrera-Jaime, Piredda, Choi), two were rated as being problematic (Mubaraki, Mohammadi), while five were considered to be at a low risk of bias (Dodd 2003, Satheeshkumar, Alkhouli, Chitapanarux, Dodd 2000). The most frequent higher risk regarding mucositis prevention was related to *outcome measurement* (50%); and the risk of the assignment effect related to intervention was 29% for studies on mucositis treatment and 29% concerning outcome data (Fig. 2a and Fig. 2b). Specifically, the assignment effect to intervention was judged as *high* risk if the analysis was performed after the post-randomization exclusion of eligible trial participants (Cabrera-Jaime et al.; Choi et al.). Alternatively, it was assessed as *Some concern* (SC) if patients and personnel were aware of the assigned treatment during the trial, but it was not reported whether there was a deviation from the intended intervention. The latter could arise due to the clinical trial context (Mubaraki et al.) or if the number of patients, who did not receive their assigned intervention, was sufficient to have a substantial impact on the results (Mohammadi et al.). The bias of omitted outcome data was assessed as a *high risk* if studies reported the occurrence of randomized patients with a high number of omitted outcome data and the analysis was performed on a restricted sample of patients (De Sanctis et al., Cabrera-Jaime et, and Choi et al.). Indeed, sensitivity analyses were not performed on these studies nor were any bias correction methods adopted. Both of these corrections would have eliminated the existing differences between intervention groups in proportion to the omitted outcome data. The bias due to randomization process was assessed as *high risk* when no form of remote or a centrally-administered method was used to allocate interventions to participants (De Sanctis et al.). The risk of bias, which was related to the effect of adhering to intervention, was assessed as *high risk* if there was a high discontinuation rate, whose effect was not examined by means of appropriate statistical methods (De Sanctis et al.). The risk of bias in outcome measurement was considered as *high risk* if the assessors were aware of the intervention received by study participants and it was not reported whether this knowledge could have influenced the outcome assessment (Piredda et al., De Sanctis et al.) Tables 4 and 5.

**Table 4:** Risk of bias evaluation for the selected RCT studies on prevention

RCT studies	Risk of bias arising from the randomization process	Risk of bias due to deviations from the intended interventions (effect of assignment to intervention)	Risk of bias due to deviations from the intended interventions (effect of adhering to intervention)	Risk of bias due to missing outcome data	Risk of bias in measurement of the outcome	Risk of bias in selection of the reported result	Overall bias
De Sanctis (2019) [19]	HR	LR	HR	HR	HR	LR	HR
Alkhouli (2021) [20]	LR	LR	LR	LR	LR	LR	LR
Chitapanarux (2018) [18]	LR	LR	LR	LR	LR	LR	LR
Piredda (2017) [17]	LR	LR	LR	LR	HR	LR	HR

Low risk (LR), some concerns (SC), high risk (HR) or not interpretable (NI)

**Table 5:** Risk of bias evaluation for the selected RCT studies on therapy

RCT studies	Risk of bias arising from the randomization process	Risk of bias due to deviations from the intended interventions (effect of assignment to intervention)	Risk of bias due to deviations from the intended interventions (effect of adhering to intervention)	Risk of bias due to missing outcome data	Risk of bias in measurement of the outcome	Risk of bias in selection of the reported result	Overall bias
De Sanctis (2019) [19]	HR	LR	HR	HR	HR	LR	HR
Cabrera-Jaime (2017) [25]	LR	HR	LR	HR	LR	LR	HR
Dodd (2003) [22]	LR	LR	LR	LR	LR	LR	LR
Mubaraki (2020) [16]	LR	SC	LR	LR	LR	LR	SC
Satheeshkumar (2010) [23]	LR	LR	LR	LR	LR	LR	LR
Alkhouli (2021) [20]	LR	LR	LR	LR	LR	LR	LR
Chitapanarux (2018) [18]	LR	LR	LR	LR	LR	LR	LR
Dodd (2000) [21]	LR	LR	LR	LR	LR	LR	LR
Piredda (2017) [17]	LR	LR	LR	LR	HR	LR	HR
Choi (2012)	LR	HR	LR	HR	LR	LR	HR
Mohammadi (2022) [26]	LR	SC	LR	LR	LR	LR	SC

Low risk (LR), some concerns (SC), high risk (HR) or not interpretable (NI)

## Discussion

Clinically, OM presents with erosive and/or ulcerative lesions, which can cause mild to severe pain. Depending on its severity, it can become a disabling condition, affecting the patient’s quality of life. Severe OM can lead to inadequate food intake and patients can, therefore, develop serious nutritional deficiencies, requiring parenteral nutrition. In addition, some of these patients suspend anti-cancer therapy, thereby affecting their chances of survival. Consequently, OM prevention and the treatment of pain, which are associated with mucositis, are crucial to the clinical management of cancer patients.

Although there is no conclusive evidence regarding the efficacy of standardized oral care for OM prevention, it is suggested by the MASCC/ISOO guidelines. Good oral hygiene is strongly correlated with positive benefits, preventing, albeit partially, infections or sepsis events during mucosae ulceration. Whilst there may be a paucity of guidelines regarding OM treatment (on account of limited evidence from high-quality, rigorous studies), SB rinses are the most frequently used agent for OM prevention and the treatment of OM-CT in patients undergoing cancer therapy [12].

NComplementary and alternative medicine, including the use of aloe-vera and propolis, have been compared to SB in order to evaluate their clinical efficacy in preventing OM. The results show that both agents are more efficient than SB in OM prevention. Propolis has a long history of medical application on account of its many biological properties, mostly due to its phenolic compounds, such as flavonoids. The following properties regarding oral mucosa diseases are widely acclaimed: immunomodulatory, antimicrobial, antifungal, anaesthetic and anti-inflammatory. However, Piredda et al. has recognized that propolis composition can be difficult to standardize, and its composition may vary across regions because it depends on the phyto-geographic characteristics of the collection sites by bees; thus, the resinous substances used in its production may be modified [17].

Similarly, aloe-vera is a natural drug, which is characterized by several properties: it promotes wound healing, it possesses anti- microbial and antioxidant properties and it eliminates free radicals. It has also been proved that aloe-vera is beneficial in treating and protecting oral tissue, particularly regarding: oral lichen planus, burning mouth syndrome, radiation-induced OM, and recurrent oral aphthous-induced stomatitis [20]. In 2016 Mansouri et al. conducted a trial involving lymphoma and leukemia patients, which demonstrated that topical applications of aloe-vera are effective in decreasing the intensity and pain of OM [29].

The efficacy of benzydamine hydrochloride (HCl) mouthwash versus SB has been evaluated in the prevention of concurrent chemoradiation-induced OM in head and neck cancer patients by Chitapanarux et al. [18]. It has already been confirmed to decrease the severity of OM in patients, who received less than 50 Gy radiation therapy [30], but to date it has not been studied in patients with concomitant chemotherapy. This RCT has demonstrated that prophylaxis with benzydamine HCl mouthwash can be more effective in reducing the severity of OM when compared to basic care with SB mouthwash due to its local anti-inflammation, analgesia, and non-specific antibacterial properties. However, no statistical differences have been reported regarding the use of *Lactobacillus brevis* CD2 lozenges and SB in preventing radiation-induced OM. The *L. brevis* CD2 production of arginine deaminase is responsible for diminishing the availability of arginine in the oral cavity, thereby reducing the production of nitrous oxide and the arginine-dependent growth of microorganisms, both of which are implicated in inflammatory processes.

Different studies have been conducted with CHX mouthwash in order to identify an effective intervention protocol for the management of OM. Dodd et al. tested three different mouthwashes: salt and SB (this combination raised oral pH and prevented the overgrowth of aciduric bacteria); CHX mouthwash 0.12% (antimicrobial activity); and a mixture of lidocaine solution, diphenhydramine hydrochloride and aluminum hydroxide suspension (aiming at promoting analgesic and anaesthetic effects); no significant differences over time for the cessation of signs and symptoms among the 3 groups were observed [21]. In 2012 Choi et al. compared the effectiveness of SB solution with CHX mouthwash. They observed that a lower percentage of patients in the SB group (25%) developed ulcerative OM, as compared to the CHX group (62,5%) There was also a delay in the onset of ulcerative OM in the SB group (16.1 vs 11.4 days) [24]. In 2017 Cabrera-Jaime et al. randomized patients to one of three treatments, consisting of SB 5% aqueous solution with: an additional dose of sodium bicarbonate 5% aqueous solution, *Plantago major* extract (already used for its anti-inflammatory properties in treating oral diseases, such as gingivitis and canker sores), or CHX 0.12%. The healing time was observed to be reduced with the double SB solution, compared to the other two rinses, but these differences were not significant [25]. Finally, in 2020, Mubaraki et al. assessed the efficacy of supersaturated calcium phosphate (introduced in 2009, a natural electrolyte solution resembling the ionic and pH balance of saliva) by comparing three groups; an existing oral hygiene protocol (a combination of 0.12% CHX gluconate, SB 3% aqueous solution and 5000 IU nystatin) was proposed alone or in association with an extra soft-toothbrush or a small amount of supersaturated calcium phosphate oral spray. The results of this study revealed no significant differences in the incidence of OM even if there was a lower degree of severity of OM in the supersaturated calcium phosphate rinse group and a significant reduction in the tolerance of CHX after chemotherapy [16].

The Californian study group, comprising Dodd et al., conducted another RCT in order to compare the efficacy of micronized sucralfate (Carafate R) mouthwash and salt & SB mouthwash for RT-induced mucositis in head and neck cancer patients. Sucralfate is cytoprotective for mucosa and, when used orally, it forms an adhesive, paste-like substance which attaches ionically to proteins in the damaged mucosa. The results have demonstrated that there is no significant difference in efficacy between micronized sucralfate and salt & SB, and that the use of the less costly salt & SB is prudent and cost-effective [22]. Satheeshkumar et al. compared triclosan mouth wash (a broad-spectrum antibacterial and anti-inflammatory agent) and SB mouth wash in radiation-induced OM. They demonstrated that triclosan mouthwash was found to be effective in reducing the severity of radiation- induced OM and it assisted in the early reversal of symptoms during the post treatment period [23]. The aim of the latest and most recent study, which was conducted by Mohammadi et al., was to evaluate the effectiveness of SB and zinc chloride mouthwashes on OM and the quality of life in patients undergoing chemotherapy. The results revealed that zinc chloride and SB mouthwashes were effective in reducing the severity of OM, and subsequently improving the quality of life of patients [26].

## Conclusion

In conclusion, the evidence presented in this systematic review is not conclusive in suggesting SB as a panacea for treating OM, as has been proposed to date by several medical specialists worldwide. The results presented in this study have revealed that the evidence for supporting SB in the treatment of OM (that is, management and prevention) were limited and of low quality. There was a paucity of RCT in the literature, in which the efficacy of SB against other medical/natural agents was compared. Those which had been analyzed had a high risk of bias due to key elements in designing an RCT. These included: errors in the randomization or lack of blinding, both of which can bias estimates of treatment effect. Moreover, there exists no gold standard of a recognized scale, with which to score OM severity, and usually patients undergoing treatment for OM are not screened by trained oral specialists. Both of these factors can affect research results, thereby inducing misleading conclusions.

The latest scientific findings regarding cancer therapies are prolonging the survival of patients but simultaneously little progress has been made regarding antineoplastic, therapy-induced OM treatment or prevention. Currently, the incidence of OM remains underestimated but it is essential to continue research in the field of OM management and prevention (using RCT to determine the true relative efficacy of the intervention) in order to provide cancer patients with an improved quality of life.

## Data Availability

The datasets used and/or analysed during the current study are available from the corresponding author on reasonable request.

## References

[1] Carrozzo M, Eriksen JG, Bensadoun RJ, Boers-Doets CB, Lalla RV, Peterson DE (2019). Oral mucosal injury caused by targeted Cancer therapies. J Natl Cancer Inst - Monogr.

[2] Pulito C, Cristaudo A, La PC, Zapperi S, Blandino G, Morrone A (2020). Oral mucositis: the hidden side of cancer therapy. J Exp Clin Cancer Res.

[3] Damascena LCL, de Lucena NNN, Ribeiro ILA, Pereira TL, Lima-Filho LMA, Valença AMG (2020). Severe oral mucositis in pediatric cancer patients: survival analysis and predictive factors. Int J Environ Res Public Health.

[4] Guimarães JR, de Carvalho LGA, Damascena LCL, Sampaio MEA, Ribeiro ILA, de Sousa SA (2021). The incidence of severe oral mucositis and its occurrence sites in pediatric oncologic patients. Med Oral Patol Oral y Cir Bucal.

[5] Jacobs S, Baggott C, Agarwal R, Hesser T, Schechter T, Judd P (2013). Validation of the Children’s international Mucositis evaluation scale (ChIMES) in paediatric cancer and SCT. Br J Cancer [Internet].

[6] Tomlinson D, Judd P, Hendershot E, Maloney AM, Sung L (2007). Measurement of oral mucositis in children: a review of the literature. Support Care Cancer.

[7] Worthington HV, Clarkson JE, Bryan G, Furness S, Glenny A-M, Littlewood A (2011). Interventions for preventing oral mucositis for patients with cancer receiving treatment. Cochrane Database Syst Rev.

[8] El Bousaadani A, Eljahd L, Abada R, Rouadi S, Roubal M, Mahtar M (2016). Actualités de la prévention et du traitement des mucites orales chez les enfants cancéreux: Recommandations pratiques. Cancer/Radiotherapie.

[9] Sung L, Robinson P, Treister N, Baggott T, Gibson P, Tissing W (2017). Guideline for the prevention of oral and oropharyngeal mucositis in children receiving treatment for cancer or undergoing haematopoietic stem cell transplantation. BMJ Support Palliat Care.

[10] Feng J, Gao M, Zhao C, Yang J, Gao H, Lu X (2022). Oral Administration of Probiotics Reduces Chemotherapy-Induced Diarrhea and Oral Mucositis: A Systematic Review and Meta-Analysis. Front Nutr.

[11] Daugėlaitė G, Užkuraitytė K, Jagelavičienė E, Filipauskas A (2019). Prevention and treatment of chemotherapy and radiotherapy induced oral mucositis. Med.

[12] Hong CHL, Gueiros LA, Fulton JS, Cheng KKF, Kandwal A, Galiti D, Fall-Dickson JM, Johansen J, Ameringer S, Kataoka T, Weikel D, Eilers J, Ranna V, Vaddi A, Rajesh V, SEO behalf of the MSG of the MA of SC in CS for OO /MASCC G pdfcolog. (MASCC/ISOO). Systematic review of basic oral care for the management of oral mucositis in cancer patients and clinical practice guidelines. Support Care Cancer. 27(10):3949–67. 10.1007/s00520-019-04848-4%0A. 10.1007/s00520-019-04848-4 31286232

[13] Maurer J (1977). Providing optimal oral health. Nurs Clin North Am.

[14] Carl W (1980). Dental management of head and neck cancer patients. J Surg Oncol.

[15] Sousa FA, Paradella TC, Koga-Ito CY, Jorge AOC (2009). Effect of sodium bicarbonate on Candida albicans adherence to thermally activated acrylic resin. Braz Oral Res.

[16] Mubaraki S, Pani SC, Alseraihy A, Abed H, Alkhayal Z (2020). The efficacy of two different oral hygiene regimens on the incidence and severity of oral mucositis in pediatric patients receiving hematopoietic stem cell transplantation: a prospective interventional study. Spec Care Dent.

[17] Piredda M, Facchinetti G, Biagioli V, Giannarelli D, Armento G, Tonini G (2017). Propolis in the prevention of oral mucositis in breast cancer patients receiving adjuvant chemotherapy: a pilot randomised controlled trial. Eur J Cancer Care (Engl).

[18] Chitapanarux I, Tungkasamit T, Petsuksiri J, Kannarunimit D, Katanyoo K, Chakkabat C (2018). Randomized control trial of benzydamine HCl versus sodium bicarbonate for prophylaxis of concurrent chemoradiation-induced oral mucositis. Support Care Cancer.

[19] de Sanctis V, Belgioia L, Cante D, la Porta MR, Caspiani O, Guarnaccia R (2019). Lactobacillus brevis CD2 for prevention of Oral Mucositis in patients with head and neck tumors: a multicentric randomized study. Anticancer Res.

[20] Alkhouli M, Laflouf M, Alhaddad M (2021). Efficacy of Aloe-Vera use for prevention of chemotherapy-induced Oral Mucositis in children with acute lymphoblastic leukemia: a randomized controlled clinical trial. Compr Child Adolesc Nurs.

[21] Dodd MJ, Dibble SL, Miaskowski C, MacPhail L, Greenspan D, Paul SM (2000). Randomized clinical trial of the effectiveness of 3 commonly used mouthwashes to treat chemotherapy-induced mucositis. Oral Surg Oral Med Oral Pathol Oral Radiol Endod.

[22] Dodd MJ, Miaskowski C, Greenspan D, MacPhail L, Shih AS, Shiba G (2003). Radiation-induced mucositis: a randomized clinical trial of micronized sucralfate versus salt & soda mouthwashes. Cancer Investig.

[23] Satheeshkumar PS, Chamba MS, Balan A, Sreelatha KT, Bhatathiri VN, Bose T (2010). Effectiveness of triclosan in the management of radiation-induced oral mucositis: a randomized clinical trial. J Cancer Res Ther.

[24] Choi SE, Kim HS (2012). Sodium bicarbonate solution versus chlorhexidine mouthwash in oral care of acute leukemia patients undergoing induction chemotherapy: a randomized controlled trial. Asian Nurs Res (Korean Soc Nurs Sci).

[25] Cabrera-Jaime S, Martínez C, Ferro-García T, Giner-Boya P, Icart-Isern T, Estrada-Masllorens JM (2018). Efficacy of Plantago major, chlorhexidine 0.12% and sodium bicarbonate 5% solution in the treatment of oral mucositis in cancer patients with solid tumour: a feasibility randomised triple-blind phase III clinical trial. Eur J Oncol Nurs.

[26] Mohammadi F, Oshvandi K, Kamallan SR, Khazaei S, Ranjbar H, Ahmadi-Motamayel F (2022). Effectiveness of sodium bicarbonate and zinc chloride mouthwashes in the treatment of oral mucositis and quality of life in patients with cancer under chemotherapy. Nurs Open.

[27] Sterne JAC, Savović J, Page MJ, Elbers RG, Blencowe NS, Boutron I (2019). RoB 2: a revised tool for assessing risk of bias in randomised trials. BMJ.

[28] Higgins JPT, Sterne JAC, Savović J, Page MJ, Hróbjartsson A, Boutron I, Reeves B ES. A revised tool for assessing risk of bias in randomized trials In: Chandler J, McKenzie J, Boutron I, Welch V (editors). Cochrane Methods. (Issue 10 (Suppl 1).).

[29] Mansouri P, Haghighi M, Beheshtipour N, Ramzi M (2016). The effect of aloe vera solution on chemotherapy-induced stomatitis in clients with lymphoma and leukemia: a randomized controlled clinical trial. Int J Commun Based Nurs Midwife.

[30] Epstein JB, Silverman SJ, Paggiarino DA, Crockett S, Schubert MM, Senzer NN (2001). Benzydamine HCl for prophylaxis of radiation-induced oral mucositis: results from a multicenter, randomized, double-blind, placebo-controlled clinical trial. Cancer.

